# ERRATUM: Atypical secretion of cortisol in Nursing professionals

**DOI:** 10.1590/1980-220X-REEUSP-2024-ER15en

**Published:** 2024-10-07

In the article “Atypical secretion of cortisol in Nursing professionals”, with the DOI: https://doi.org/10.1590/S0080-623420150000700016, published by the journal: “Revista da Escola de Enfermagem da USP, volume 49 (spe) of 2015, p. 107–114, on page 114:

**Where it was written:**

## Financial Support:

Fundação de Amparo à Pesquisa do Estado de São Paulo – FAPESP. Process number: 2013/01637-2.

**Now read:**

## Financial Support:

Fundação de Amparo à Pesquisa do Estado de São Paulo – FAPESP. Process number: 2013/01637-2. This study was financed in part by the Coordenação de Aperfeiçoamento de Pessoal de Nível Superior – Brasil (CAPES) – Finance Code 001.

