# Supplementary material for: ERRATUM: Atypical secretion of cortisol in Nursing professionals
Source: Rev Esc Enferm USP. 2024 Oct 7;58:e2024ER15. doi: 10.1590/1980-220X-REEUSP-2024-ER15en (PMC11524411; doi:10.1590/1980-220X-REEUSP-2024-ER15en)
Supplement: Translation

# Translation: ERRATA: Padrão atípico de secreção de cortisol em profissionais de Enfermagem

No artigo “Padrão atípico de secreção de cortisol em profissionais de Enfermagem”, com número de: DOI: https://doi.org/10.1590/S0080-623420150000700016, publicado na Revista da Escola de Enfermagem da USP, volume 49 (Esp) de 2015, p. 109–116, na página 116:

**Onde se lia:**

## Apoio Financeiro:

Fundação de Amparo à Pesquisa do Estado de São Paulo (FAPESP). Processo n. 2013/01637-2.

**Leia-se:**

## Apoio Financeiro:

Fundação de Amparo à Pesquisa do Estado de São Paulo (FAPESP). Processo n. 2013/01637-2. O presente trabalho foi realizado com apoio da Coordenação de Aperfeiçoamento de Pessoal de Nível Superior – Brasil (CAPES) – Código de Financiamento 001.
